# Adherence and Compliance with Endocrine Treatment After Primary Breast Cancer Treatment: A Cross-Sectional Qualitative Study

**DOI:** 10.3390/medicina61112055

**Published:** 2025-11-18

**Authors:** Odhran Cosgrove, Sadaf Zehra, Dinesh Kumar Thekkinkattil

**Affiliations:** 1 Lincoln Medical School, University of Nottingham, Ross Lucas Medical Sciences Building, Lincoln LN6 7FS, UK; odhran.cosgrove@nhs.net; 2 Lincoln Breast Unit, Lincoln County Hospital, United Lincolnshire Teaching Hospitals NHS Trust, Lincoln LN2 5QY, UK; sadaf.zehra@nhs.net

**Keywords:** breast cancer, adherence, endocrine therapy, compliance, questionnaire

## Abstract

*Background and Objectives:* Breast cancer is the most common cancer in women, with approximately 80% being oestrogen receptor positive, necessitating adjuvant endocrine therapy (AET) to reduce recurrence. Treatment adherence is crucial, yet 10–50% of patients take incorrect doses or discontinue therapy, which is associated with a 20% increase in mortality. AET may also impact bone health. This study aimed to explore patients’ beliefs about endocrine treatment, investigate how perceptions of medication risk and benefit affect adherence, and assess changes in bone mineral density (BMD) during therapy. *Materials and Methods:* A cross-sectional mixed-method study was conducted. One hundred patients diagnosed with oestrogen receptor-positive breast cancer in 2020 were sent the Beliefs about Medicines Questionnaire–Adjuvant Endocrine Therapy (BMQ-AET) and 101 semi-structured telephone interviews were completed. Initial and most recent Dual-Energy X-ray Absorptiometry (DEXA) scans were compared to assess changes in BMD. *Results:* The questionnaire response rate was 55% (n = 55). Forty-nine patients returned the postal paper survey and six patients responded via QR code. One hundred and one patients participated in semi-structured telephone interviews. Of the total cohort, 91.7% were adherent to AET, while 13 patients (8.3%) were non-adherent. Non-adherent patients had significantly lower BMQ-AET Necessity scores (mean 12.08 vs. 19.22; median 12 vs. 20; *p* < 0.001) and higher Concerns scores (mean 17 vs. 13.46; Median 17 vs. 13; *p* = 0.002). The most common reasons for non-adherence were joint pain and reduced quality of life (58%), highlighting a need for additional support in managing side effects. Among the participants with suitable DEXA data, the majority (54.2%) demonstrated an increase in BMD over time. *Conclusions:* This study demonstrates high adherence to AET, with non-adherent patients showing lower perceived necessity and greater concern about treatment. These findings emphasise the importance of addressing patient beliefs to enhance adherence. The observed improvements in BMD suggest that proactive bone health management, alongside AET, may mitigate expected declines, challenging conventional assumptions regarding therapy-related bone loss.

## 1. Introduction

### 1.1. Background

Breast cancer (BC) is the most common cancer among women in the United Kingdom and worldwide, accounting for around 15% of all new cancer cases between 2017 and 2019 [1,2]. Primary management usually involves surgery, either breast-conserving surgery, mastectomy, and/or axillary lymph node surgery, often followed by radiotherapy to reduce recurrence risk [3]. For oestrogen receptor-positive (ER-positive) tumours, which comprise approximately 80% of all BC cases, adjuvant endocrine therapies (AET) such as tamoxifen or aromatase inhibitors (AIs) are recommended to reduce recurrence and improve survival [4].

### 1.2. Endocrine Therapy and Breast Cancer

Oestrogen drives breast cancer cell proliferation through receptor-mediated signalling that promotes growth and suppresses apoptosis [5]. These mechanisms underpin the use of AET, which targets oestrogen receptor pathways to reduce the hormonal stimulation of tumour cells [6].

### 1.3. Current Endocrine Treatments

Tamoxifen acts as a selective oestrogen receptor modulator with anti-oestrogenic effects in breast tissue [7]. In contrast, AIs inhibit the aromatase enzyme, blocking the conversion of androgens to oestrogens [8]. The choice of management depends on menopausal status. AIs are the first-line treatment for postmenopausal women and are shown to reduce breast cancer mortality by 30% over 15 years compared with tamoxifen [9]. In premenopausal women, tamoxifen is used alone or in combination with gonadotropin-releasing hormone (GnRH) agonists to achieve reversible ovarian suppression, allowing transition to AI therapy after menopause [10].

### 1.4. Efficacy of AET

The efficacy of AET in reducing recurrence and mortality is well established. The Early Breast Cancer Trialists’ Collaborative Group found that 5 years of tamoxifen reduced breast cancer deaths by 31% [11]. Another study showed that tamoxifen halved recurrence risk in the first four years and reduced it by a third between years five and nine [12]. The ATAC trial demonstrated superior efficacy and fewer side effects with anastrozole compared with tamoxifen in postmenopausal women [13]. Extended AET beyond five years further reduced recurrence and mortality, with an approximately 50% decline in breast cancer mortality during the second decade after diagnosis [14].

### 1.5. Adherence and Compliance

Despite its proven benefits, long-term adherence to AET is suboptimal. Adherence reflects an active choice to follow treatment, while compliance refers to passive following of medical advice [15,16]. Adherence typically declines over time, with rates dropping from approximately 80% in the first year to 55% by the fifth [17]. Non-adherence can increase mortality risk by nearly 50% [18,19]. Understanding the factors influencing persistence is therefore essential.

### 1.6. Determinants of Non-Adherence

AET adherence is multifactorial, affected by demographics, side effects, and psychosocial factors. Younger (<45) and older (>80) patients are often less adherent due to fertility concerns or comorbidities [20,21]. Polypharmacy and limited healthcare support can also reduce adherence [22,23]. Side effects, particularly arthralgia, hot flushes, and menopausal symptoms, affect up to 94% of patients and significantly impact quality of life and persistence [24]. While education and supportive communication from healthcare professionals can mitigate these effects, patient beliefs about medication necessity and concerns about long-term side effects also play a major role [25,26,27,28]. Higher education is generally associated with improved adherence, likely due to a greater understanding of the treatment’s importance [28].

### 1.7. AET and Bone Health

AET can significantly influence bone mineral density (BMD), depending on drug type and menopausal status. In postmenopausal women, tamoxifen acts as a partial oestrogen agonist, reducing fracture risk by 32% compared with placebo [29]. Conversely, in premenopausal women, tamoxifen may cause bone loss, and AIs are consistently linked to increased bone resorption and fracture risk [30]. AIs combined with ovarian suppression result in the most profound bone loss [29]. Consequently, bone health monitoring using Dual-Energy X-ray Absorptiometry (DEXA) is recommended, alongside preventive strategies such as bisphosphonates and calcium and vitamin D supplementation [30,31].

### 1.8. Studies Aims

While the efficacy of AET is well established, adherence remains inconsistent, and its impact on bone health in real-world settings is not fully understood. Exploring both factors concurrently may provide insights into how patient beliefs, side effects, and concurrent interventions influence treatment persistence and outcomes. This cross-sectional mixed-method study aims to (1) assess adherence to AET among breast cancer patients, (2) explore patient beliefs and factors influencing non-adherence, and (3) evaluate the effect of AET on bone mineral density.

## 2. Methodology

### 2.1. Questionnaire

#### 2.1.1. Participants

The participants were patients diagnosed with BC who had received treatment with curative intent within the high volume breast centre in a district general hospital in the United Kingdom in 2020 and had commenced AET, allowing for adequate duration of treatment.

Inclusion Criteria: Patients diagnosed with oestrogen receptor positive early and locally advanced breast cancer who had treatment with curative intent and were commenced on adjuvant endocrine treatment. Patients were chosen for the questionnaire survey according to their chronological order of diagnosis in our database. The first 100 eligible patients were chosen for the questionnaire survey and the next 101 eligible patients were chosen for the semi-structured interview.

Exclusion Criteria: Patients who had advanced ER-positive breast cancer and were treated with palliative intent were excluded from our study. Patients who were diagnosed with ER negative breast cancers were also excluded from our study.

#### 2.1.2. Questionnaire Creation

An extensive literature search was conducted using PubMed and Google Scholar with terms such as “medication compliance,” “endocrine therapy adherence,” and “breast cancer medication compliance.” Three validated questionnaires were identified as relevant: the ASK-12 Survey, General Medication Adherence Scale (GMAS), and the Beliefs about Medicines Questionnaire (BMQ) [32,33] The BMQ had previously been adapted for adjuvant endocrine therapy (BMQ-AET) to assess adherence in women with breast cancer [4]. After evaluating each survey’s strengths and limitations, the BMQ-AET was selected as the most suitable for the study’s aims.

To address gaps in existing questionnaires, particularly regarding the degree of adherence, age at diagnosis, education level, side effect profile, and suggestions for improvement, custom questions were added. These included Likert-scale items (5 = Strongly Agree, 1 = Strongly Disagree), yes/no items, and optional open-ended responses to allow participants to elaborate.

#### 2.1.3. Data Collection

One hundred surveys were distributed in November 2024, accompanied by a cover letter explaining the study’s purpose and an estimated completion time of 10 min. To maximise participation, participants could complete the survey either on paper, returned via prepaid envelope, or digitally via a QR code or web link provided on the cover letter. This mixed-mode approach aimed to enhance response rates within the study timeframe. Due to a limited timeframe, the response window for the questionnaire was 2 months.

### 2.2. Semi-Structured Telephone Interviews

#### 2.2.1. Participants

A further pool of participants was selected from the database of patients that been diagnosed with ER-positive BC in 2020 who were treated with curative intent and prescribed AET. Selection was purely based on the choronological order of date of diagnosis of their cancer.

#### 2.2.2. Study Design

The semi-structured telephone interviews mainly used questions from the survey. However, the interviewer allowed more open-ended responses from participants. The use of semi-structured telephone interviews was to maximise response while also gauging the participants’ feelings behind their response.

#### 2.2.3. Data Collection (Semi-Structured Interview)

The semi-structured telephone interviews occurred at the end of October and throughout November 2024. The interviewer explained the reasoning and purpose of the interviews before any questions were asked and consent was gained. The semi-structured aspect of the interviews allowed for more flexible questioning to gain further insight into the participants’ beliefs or burdens behind the treatment.

### 2.3. DEXA Scan Comparison

#### 2.3.1. Patient Cohort

Of those participants who completed the questionnaires or semi-structured interviews, DEXA scan results were reviewed retrospectively. Patients who had not attended any DEXA scan or only attended the initial DEXA scan were discarded from the comparison; however, the bone mineral density (BMD) on the baseline DEXA was recorded.

#### 2.3.2. Data Collection

In December 2024, the participants’ most recent DEXA scans were compared against their initial DEXA scans in order to assess any change in BMD after taking AET. These comparisons were conducted retrospectively, based on routinely collected clinical data to explore potential long-term skeletal effects of endocrine therapy. The institutional guideline for follow-up DEXA scans is every two years if the patient is on AI and not on any adjuvant bisphosphonates as part of their cancer treatment. As per departmental protocol, patients who had adjuvant bisphosphonates as part of their adjuvant treatment did not have a DEXA scan till they finished their adjuvant treatment

#### 2.3.3. Data Analysis

Questionnaire and semi-structured interview responses were manually entered into Microsoft Excel. Participants were grouped by adherence to AET (adherent vs. non-adherent) based on their response to Question 1 (see Appendix A). Due to unequal group sizes (143 adherent, 13 non-adherent), results were presented as percentages rather than raw counts, with trends visualised using bar and pie charts.

Responses from the BMQ-AET were converted into ordinal numerical values (5 = Strongly Agree, 1 = Strongly Disagree) and separated into Necessity and Concerns subscales. Given the ordinal nature of the data, medians and interquartile ranges (IQRs) were used to summarise responses. Non-parametric analysis was performed using the Mann–Whitney U test to compare adherent and non-adherent groups. Effect sizes (r) were calculated and interpreted according to conventional thresholds (0.1 = small, 0.3 = medium, 0.5 = large). Confidence intervals (CIs) were included for the main outcome measures, particularly the difference between adherent and non-adherent BMQ subscale scores. The *p*-values were calculated for each subscale and visualised with box-and-whisker plots. All statistical analyses were conducted using numiqo: Online Statistics Calculator: numiqo e.U.Graz.Austria (URL https://numiqo.com).

#### 2.3.4. Thematic Analysis

A thematic analysis of comments from patients was carried out, considering responses to questions about the reason for stoppage and, if they stopped taking medication, what could have done better to help them continue with the medication.

For DEXA scan comparisons, baseline BMD and subsequent changes were recorded. Bar and pie charts were used to display BMD changes and the proportion of patients in each category.

### 2.4. Ethical Considerations

Participants of both the questionnaire and the semi-structured telephone interview were informed of the optional nature of taking part, and it was highlighted to them that they could retract their data and withdraw at any point. Confidentiality was of the utmost importance within the study. Therefore, the anonymity of the data collected was emphasised to the participants. The study was approved by the Institutional Governance department and the Patient Experience department and registered as a quality improvement project.

## 3. Results

### 3.1. Response Rates

**Figure 1:**
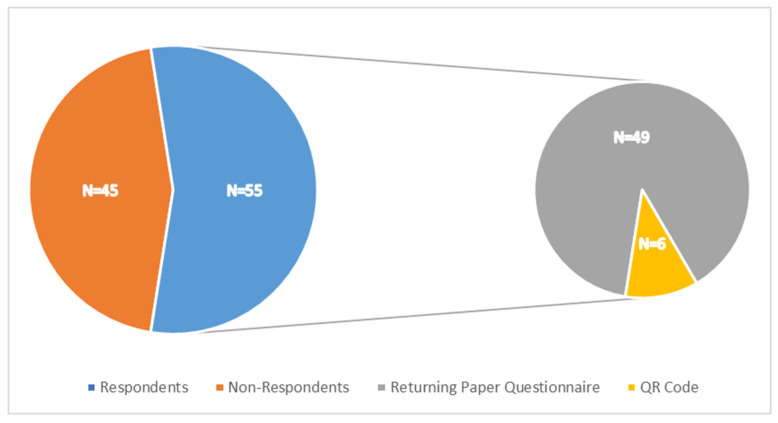
Response Rates of Distributed Postal Questionnaire.

One hundred patients were contacted via post using the BMQ-AET questionnaire (Necessity and Concerns subscales) alongside custom-made questions. The response rate for the postal questionnaires was 55 (55%), of which 49 responded by returning the paper questionnaire by post and six responded using the QR code (Figure 1). A further 101 patients were contacted for a semi-structured interview via the telephone. Therefore, a total population of 156 patients were included within this study.

### 3.2. Demographics

**Figure 2:**
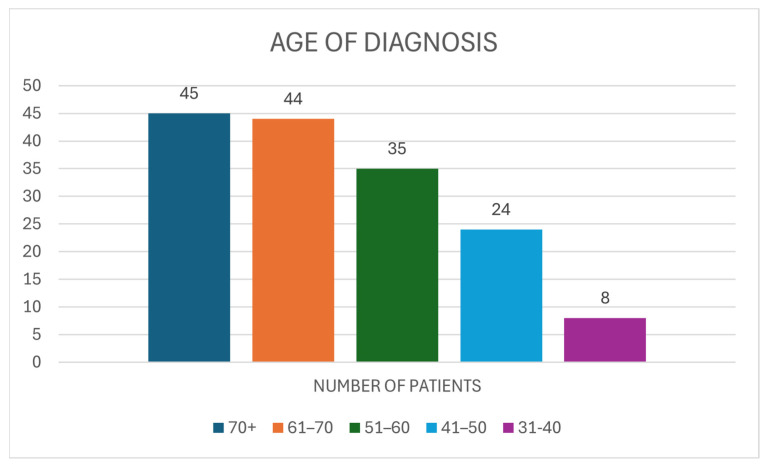
Distribution of Age of Diagnosis of Patients (N = 156).

The age of diagnosis of breast cancer within the population was shown to be older, with 28.88% (N = 45) within the 70+ age group. The subsequent most prevalent age group was between 61 and 70, as a further 28.21% (N = 44) belonged within this category. A total of 23.07% (N = 35) of the patients were between the ages of 51 and 60, as opposed to the 41–50 age group which consisted of 15.38% (N = 24) of the respondents. Lower numbers of the population occurred in younger age groups, as only 5.13% (N = 8) were 31–40 years of age at diagnosis (Figure 2).

Participants were asked a question about the highest level of education they had achieved. The most abundant education level was O-Levels/GCSE’s, with 46 (29.49%) of patients completing them as their highest educational level. Patients who answered ‘No Formal Qualifications’ made up 19.23% (N = 30) of the cohort, and those who chose not to specify their education levels made up a further 21.15%% (N = 33). Graduates included 26 participants (16.67%). The next group consisted of patients who had completed A-Levels as their highest level of education, consisting of ten individuals (6.41%). The remaining individuals, 11 (7.05%), answered the “Other” section, which included certifications such as diplomas and apprenticeships.

**Figure 3:**
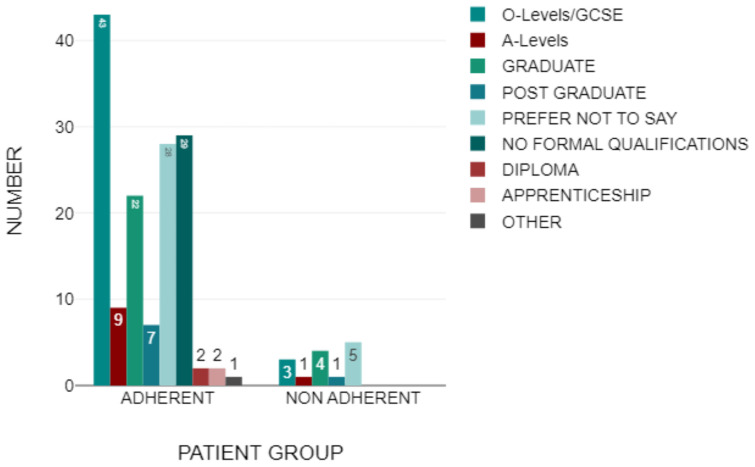
The Highest Level of Education Achieved by Participants of the Study.

The breakdown of the education level relating to adherence within this trial is seen in Figure 3 but will be examined in greater detail later in this section.

### 3.3. Adherent Group

**Figure 4:**
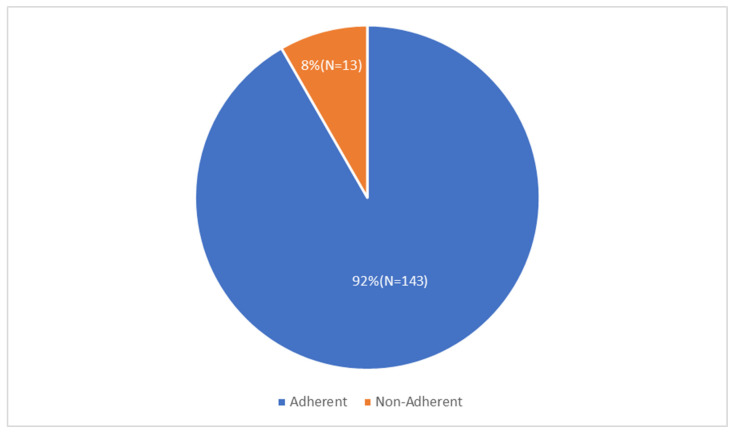
Proportion of Patients within this study who were Adherent and non-adherent to their AET.

Of the total 156 participants, 143 (91.67%) were found to be adherent to AET after BC. (Figure 4).

**Figure 5:**
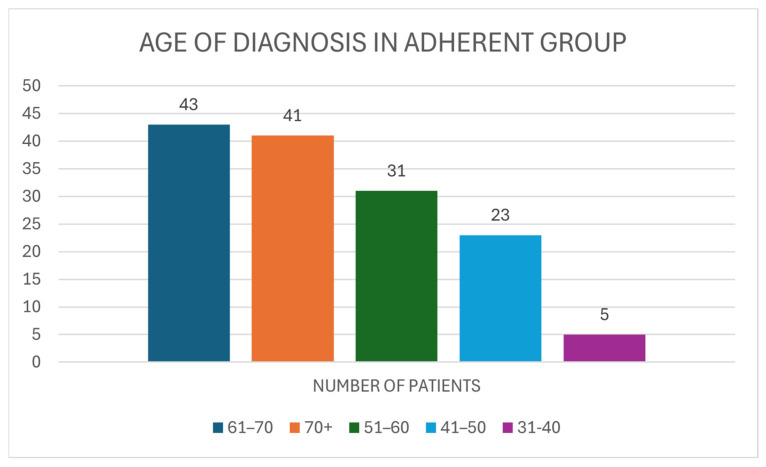
The age of diagnosis for BC of individuals within the adherent group.

Within the adherent group, the age at diagnosis of participants can be seen. The most populous age within the adherent group was 61–70 with 43 (30.07%) patients. Similarly, 41 (28.67%) of the participants were aged 70 and above. Patients who were diagnosed with BC between the ages of 51 and 60 made up 21.68% (N = 31) of the population. Twenty-three (16.08%) of those adherents with AET were diagnosed between the ages of 41 and 50. Finally, the age group with the lowest number of participants within the adherent group was 31–40, having five participants (3.5%). This is shown in Figure 5.

**Figure 6:**
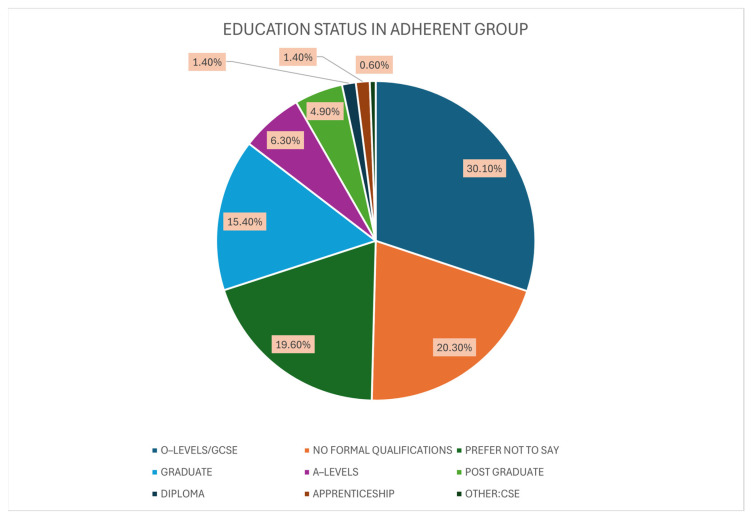
The Highest Level of Education Achieved within the Adherent Group.

Next to be tested was the education status within the adherent group. O-Levels/GCSE had the highest percentage, 30.1%, within the adherent group (N = 43). The second most popular group within the adherent group included those with no formal qualifications, amounting to 29 patients (20.3%). Similarly to the previous, 28 individuals (19.6%) chose the “Prefer Not to Say” option. 15.4% of the adherent group were graduates, with 22 patients. A further nine patients (6.29%) completed A-Levels as the highest form of formal qualifications and seven more (4.9%) achieved postgraduate status. The “Diploma” and “Apprenticeships” educational groups consisted of two individuals, both accounting for 1.4% of the adherent group each. The final 1 individual stated CSE’s was their highest form of qualification, representing 0.699% of the adherent group (Figure 6).

### 3.4. Non-Adherent Group

Non-adherence to AET within the 156 participants was shown in 13 (8.33%) of the participants of the study. This was highlighted previously in Figure 4.

**Figure 7:**
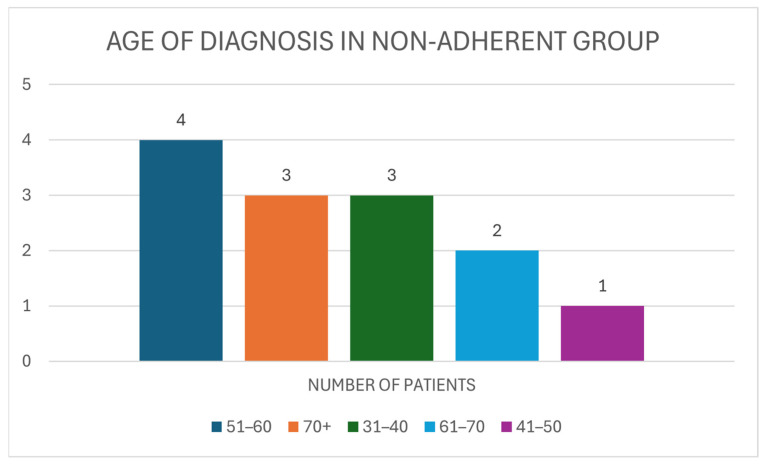
The age of diagnosis for BC of individuals within the non-adherent group (N = 13).

The age at BC diagnosis within the non-adherent group differs to that of the adherent group, as the ages of 51–60 accounted for the most populous group, making up 30.77% (N = 4) of those non-compliant with AET. Three individuals who were aged 70 or over at diagnosis made up 23.08% of the non-adherent group. These numbers are identical for the age range of 31–40 years. Of the 13 patients in this group, 15.38% (N = 2) fell within the 61–70 age category and the final 1 individual (7.69%) was between the ages of 41 and 50 at diagnosis (Figure 7).

**Figure 8:**
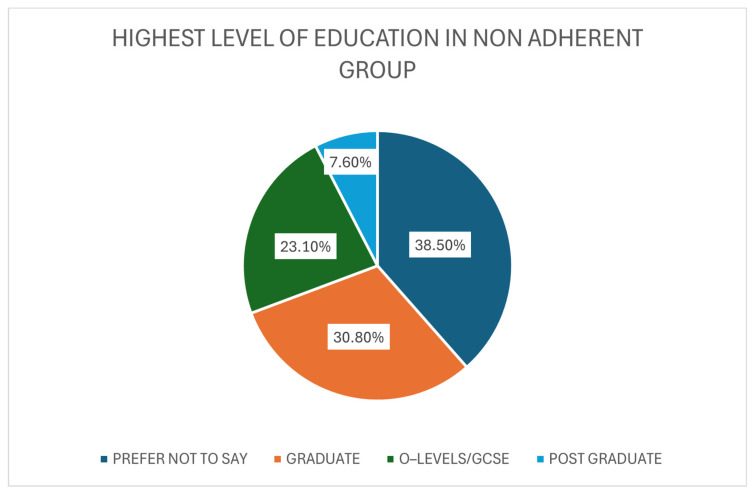
The Highest Level of Education Achieved within the Non-adherent Group (N = 13).

Education status within the non-adherent group was also measured. The main proportion of responses of the group was “Prefer Not to Say”, which consisted of five participants (38.5%). Of the individuals who specified their education status, 30.8% (N = 4) held graduate level qualifications. Three patients stated that they held O-Levels/GCSE’s as their highest form of formal qualification, accumulating to 23.1% of the non-adherent group. Finally, 1 individual (7.69%) held a postgraduate level of education (Figure 8).

**Figure 9:**
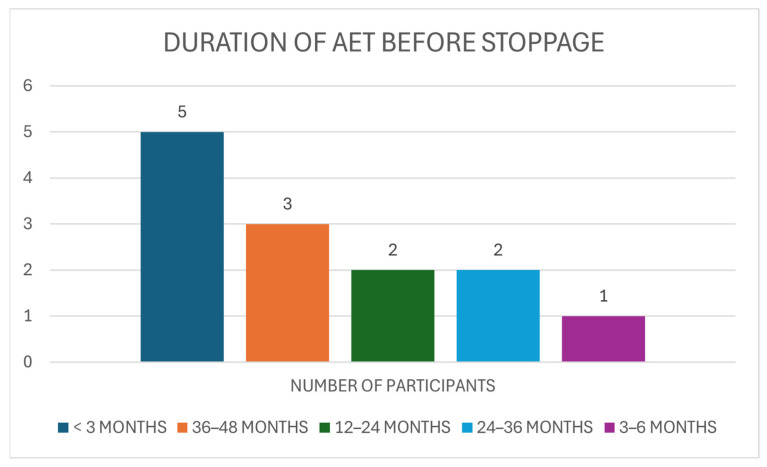
The Duration in Months of AET Treatment before stoppage within Non-adherent Group.

Patients who stated that they were non-compliant with their AET were asked about the duration of treatment (in months) before stopping. Five (38.46%) of those asked stated they were on AET for less than 3 months. Three patients (23.08%) had a duration of treatment lasting 36–48 months. A further two patients (15.38%) reported that they took 24–36 months of being on AET before discontinuation. The duration of 12–24 months is what it took for two patients (15.38%) to discontinue AET. One individual (7.69%) was on AET for 3–6 months, and one individual (7.69%) took 6–12 months before stoppage. No patients reported stopping after 6–12 months (Figure 9).

The reasoning behind the termination of AET adherence was asked. Of the 13 within the non-adherent group, 30.77% (4 individuals) stated that it was due to side effects consisting of both joint pain and poor quality of life. Furthermore, an additional 3 (23.08%) said their AET discontinuation was due to poor quality of life alone. Two patients (15.38%) admitted that isolated joint pain was the reasoning behind stoppage of AET. One patient (7.69%) stopped medication due to wanting to start a family. Menopausal symptoms were the reasoning behind another (N = 1) patient’s cessation of AET, accounting for 7.69%. A combination of brain fog and insomnia led an additional individual (7.69%) to stop AET. The final participant within the 13 of the non-adherent group stated that joint pain alongside hot flushes led to non-adherence of the treatment. Overall, seven out of the 13 participants (53.85%) within the non-adherent group had to stop the medication due to joint pain.

The 156 participants of the study were asked what improvements could have been made to support them while taking the medication. This question’s qualitative nature allowed for patients to expand their answers. Seventy of the participants (44.87%) answered that the question was not applicable. Of the responses, 26 (16.67%) included that better guidance on how to deal with side effects would have helped. Similarly, 25 (16.03%) responses included emotional support as a service that would have been of benefit to them while taking the medication. Twenty-three (14.74%) answers included the need for more information about the medication as a gap in the support of individuals on AET. Help with managing multiple medications was present in 7 (4.49%) of the answers for participants asked this question. Twelve (7.69%) answers contained “Other”, in which participants shared experiences regarding communication and the attitudes of their healthcare professionals.

### 3.5. BMQ-AET Score Comparison

The necessity subscale of the BMQ-AET was utilised to assess the patients’ beliefs about how necessary AET is. A comparison of the non-adherent group (n = 13) against the adherent group (n = 143) was conducted. Table 1 shows the results.

Following this, a Mann–Whitney U Test was performed. The test revealed a statistically significant difference between the BMQ-AET specific necessity score for the adherent group and the BMQ-AET Necessity score for the non-adherent group (U = 75, z = −5.51) The asymptomatic *p*-value was <0.001, with the exact *p*-value equally <0.001. The median values were increased in the adherent group as opposed to the non-adherent group, indicating a medium effect size (r = 0.44). Therefore, at a 5% significance level, the null hypothesis is rejected.

**Figure 10:**
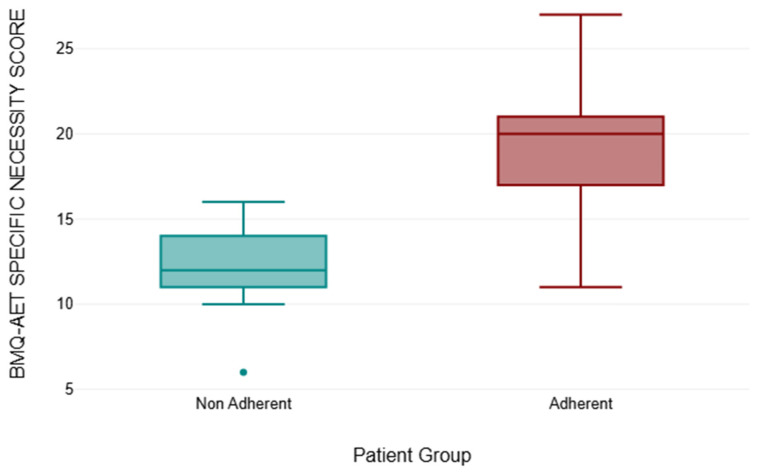
The Comparison between BMQ-AET Necessity Score between the Non–Adherent and Adherent Group (*p* =< 0.001, Mann–Whitney U Test r = 0.44).

The results from the BMQ-AET Necessity Score Comparison can be seen in Figure 10.

Alternatively, the concerns subscale of the BMQ-AET was used to assess the patients’ concerns over AET. Another comparison between the non-adherent group (N = 13) and the adherent group (N = 143) was made. Table 2 shows the results.

A sum of ranks and a mean rank was calculated for both the non-adherent and the adherent group of participants using the BMQ-AET specific concerns score. From the non-adherent group (N = 13), the sum of ranks equalled 1497.50, therefore a mean rank of 115.19 was deduced. The adherent group (N = 143) had a sum of ranks amounting to 10,748.50, resulting in the mean rank of 75.16. The exact *p*-value is 0.002. This is another way of calculating the *p*-value, often used for small sample sizes. It is slightly higher than the asymptotic *p*-value, also indicating a significant result at the 5% significance level.

The r value is 0.25. This is a measure of effect size and indicates the magnitude of the difference between groups. The value of 0.25 suggests a small effect size.

In summary, the Mann–Whitney U test results suggest that there is a small effect size in the difference between non-adherent and adherent, with the non-adherent group likely having higher values. The difference is statistically significant at a 5% significance level.

**Figure 11:**
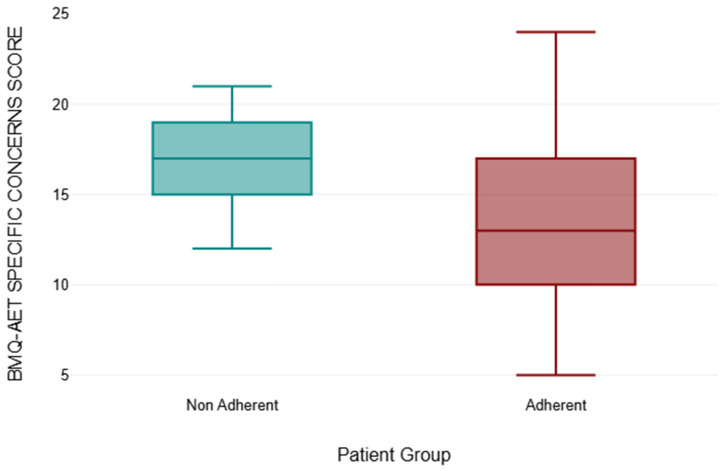
The Comparison between BMQ-AET Concerns Score between the non-adherent and adherent Group (*p* = 0.002, Mann–Whitney U Test, r = 0.25).

The results and distribution of results of the comparison between the BMQ-AET Concerns score between the non-adherent and adherent groups can be seen in Figure 11.

### 3.6. Thematic Analysis

A thematic analysis was carried out of responses from participants for questions about reasons for stopping medication, and if they stopped what could have done better to help them to continue with the prescribed medication. This identified six interconnected themes reflecting barriers and facilitators of adherence to adjuvant endocrine therapy among patients.

#### 3.6.1. Theme 1: Treatment Burden and Side Effects

Nearly all participants reported side effects from endocrine therapy. Joint pain, menopausal symptoms, fatigue, and sleep disturbances were most frequently cited challenges. Several participants described their life as ‘ruined’ or ‘worse than menopause’ suggesting that the treatment’s perceived burden outweighed its benefit for some. Nonetheless, the majority demonstrated resilience, continuing treatment despite discomfort. Some of the illustrative extracts were as follows: “Joint pain, menopausal symptoms, poor quality of life-ruined my life, felt even worse than menopause”. “Poor quality of life, tired and achy”.

#### 3.6.2. Theme 2: Psychological and Emotional Impact

Participants described psychological distress, mood swings, anxiety, depression, and emotional exhaustion linked to both treatment and the daily reminder of the cancer. The therapy’s constant presence (a daily reminder of what it is for) reinforced cancer-related anxiety and hindered emotional recovery in some of the participants. Body image issues including weight gain and hair loss further reduced self-esteem and the motivation to adhere. Some of the quotes from patients were as follows: “makes me very irritable so limits social life”, “side effects of tamoxifen made me depressed”.

#### 3.6.3. Theme 3: Information and Communication Gap

Some participants expressed dissatisfaction with the level of information provided about endocrine therapy. Patients often lacked clarity on the rationale, duration, and management of side effects. Some described confusion due to inconsistent explanations about treatment efficacy and benefits. The absence of transparent, ongoing communication contributed to feelings of uncertainty and at times disengagement from treatment. Some of the comments from patients were as follows: “I had little guidance when given the medication”, “[I] wish the oncologist was truthful on benefits and provided a little more clarity”.

#### 3.6.4. Theme 4: Emotional Support and Continuity of Care

A recurring theme was the need for emotional support and continuous care throughout endocrine treatment. Participants valued empathic relationships and regular follow up. In contrast, feeling ‘passed around’ or lacking a named nurse led to frustration. Some participants attributed this to the impact of the COVID-19 pandemic, which disrupted face-to-face care and support. Patients who received consistent emotional support reported greater satisfaction and stronger adherence. Some of the comments by participants were: “Felt I was passed around a little bit-felt if a named nurse could coordinate”.

#### 3.6.5. Theme 5: Systemic and Logistical Barriers

A subset of participants cited external, system level challenges, such as difficulty in obtaining medication, poor coordination between services, or pandemic-related isolation. These barriers compounded the personal burden of side effects. Such systemic issues highlight that adherence is not solely patient-dependent but also shaped by structural factors. Quotes by the participants included: “issue finding a pharmacy location where I can get the endocrine treatment”, “Didn’t have any face-to-face consultation after surgery”.

#### 3.6.6. Theme 6: Resilience and Trust in Clinicians

Despite the many challenges described, several participants expressed strong trust in their clinicians and determination to adhere. Phrases like ‘wouldn’t stop’, ‘would only stop on doctor’s advice’ and ‘take what the doctor gives’ reflect high faith in medical authority and a strong sense of responsibility. This theme underscores the potential role of clinician-patient relationships as protective factors supporting adherence even amid adverse effects.

Overall adherence to endocrine therapy emerges as a multifactorial process shaped by:

The physical burden of treatment

Emotional resilience

Clarity of communication

The level of social and systemic support available.

Patients who felt informed and supported and trusted their clinicians were more likely to persevere despite side effects. Conversely, insufficient guidance and poor continuity of care often led to non-adherence or consideration of discontinuation. Some of the comments were as follows: “would only stop on doctor’s recommendation”, “would never stop in spite of symptoms”.

### 3.7. DEXA Scan Comparison

**Figure 12:**
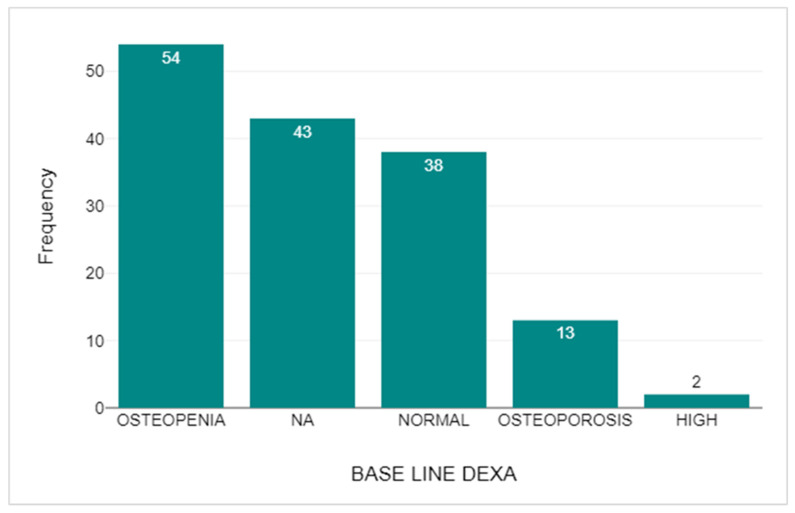
The Number of Participants with various BMD on the baseline DEXA scan.

In 156 participants, DEXA scans were compared. For the initial baseline DEXA, six participants (3.85%) were invalid and withdrawn. This left a pool of 150 (96.15%) suitable patients to compare. Of these, 54 (34.62%) individuals’ baseline DEXA showed evidence of osteopenia. A further 43 (27.56%) participants were deemed not applicable for a baseline DEXA scan as these patients were on adjuvant bisphosphonates for their cancer treatment and as per institutional protocol, DEXA scans were not carried out for these patients. Patients who had normal BMD within the baseline DEXA scan amounted to 38 (24.36%). Thirteen participants (8.33%) were deemed osteoporotic, and the final 2 participants (3.85%) had a high BMD within their baseline DEXA scan. This is shown in Figure 12.

**Figure 13:**
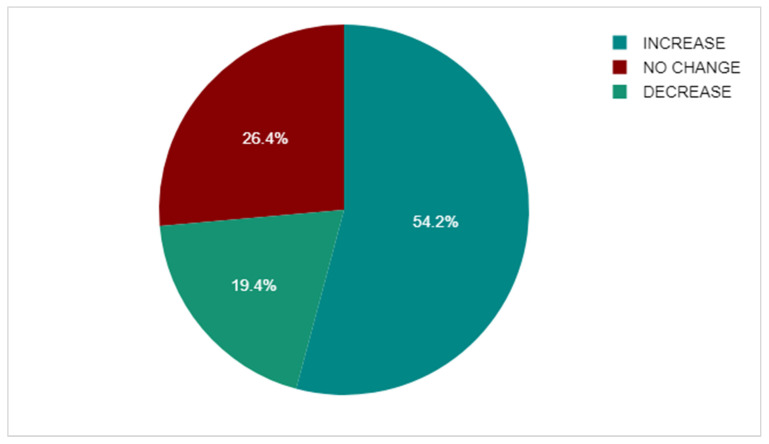
The Comparison of BMD on follow up DEXA against baseline DEXA scan.

The baseline DEXA scans were then compared to the participant’s most recent scan. Eighty-two participants (53.25%) were deemed invalid for BMD comparison on their follow up DEXA scans, leaving a population of 72 (46.75%) to compare. Of these 72, an increase in BMD was seen either at the hip or the spine in 39 patients (25.32%). There was no change observed in BMD on the follow up DEXA scan for 19 individuals (12.34%). Finally, 14 participants (9.09%) were shown to have a decrease in BMD on comparison with their follow up DEXA scan (Figure 13). It should be noted that patients with osteopenia or osteoporosis at their baseline DEXA scans commenced bone-protective therapies, including calcium, vitamin D, and bisphosphonates, according to guidelines, which may have contributed to changes in BMD. Only the trend in BMD was assessed in follow up scans, and no formal statistical comparison was made between the T scores and percentage changes in BMD between the baseline and follow up scans. The observed increase in BMD may reflect the effects of bone-protective therapy rather than AET itself.

## 4. Discussion

This study found high adherence to AET, with 91.67% of patients taking it as prescribed, contrasting with previous literature reporting lower adherence in BC patients [34]. Contributing factors may include demographics, social desirability bias, and educational level, with 52.6% (N = 82) obtaining O-Levels or higher [35,36]. Access to a Cancer Nurse Specialist Helpline and routine holistic needs assessments 12 months post-diagnosis likely supported adherence by providing guidance and information. Notably, adherence remained high during the fourth and fifth year of AET, exceeding expectations from previous studies that reported declining adherence over longer periods [12]. This again may well be due to the fact that our patients had access to continued support from their cancer nurse specialist throughout their course of treatment, and could get advice and referral to specialists if there were any needs for support and guidance.

Participants were typically older at BC diagnosis, consistent with age as a primary risk factor. However, non-adherent patients were generally younger, most commonly 51–60 years, compared to 61–70 in the adherent group. This aligns with prior studies showing that younger patients are less likely to adhere to AET, although some evidence suggests that adherence is influenced more by individual beliefs than age [37].

Education levels varied within the cohort, reflecting the general population [37]. While most adherent participants had O-levels/GCSEs, graduates were more common in the non-adherent group, contrasting prior studies that link higher education with improved AET adherence [38]. However, the small non-adherent group (N = 13) limits the robustness of this comparison.

In the non-compliant group, most participants had been on AET for less than 3 months, with no clear trend in duration before discontinuation. Side effects were the primary reason for stopping, consistent with prior research indicating that around 70% of AET patients experience side effects [37]. Joint pain was most common (53.85%), followed by menopausal symptoms, hot flushes, brain fog, and insomnia, all negatively affecting quality of life and adherence [37]. One participant discontinued due to fertility concerns, highlighting the need for guidance for premenopausal patients. Short-term cessation for conception does not appear to increase recurrence risk.

Most participants (44.9%) reported no additional support was needed while taking AET. Guidance on managing side effects (16.7%), emotional support, and clear medication information were identified as key facilitators of adherence [37,38]. Support from healthcare professionals also enhanced adherence, particularly for older adults managing multiple medications, consistent with existing research [37,38].

BMQ-AET analysis showed non-adherent participants had lower perceived necessity of AET (median = 12 vs. 20) and higher concerns about side effects (median = 17 vs. 13), consistent with the literature linking beliefs and adherence [4,37,38]. While the adherent group demonstrated more variability in responses, the overall pattern indicates that addressing patient concerns early may support adherence. These findings highlight associations rather than causal effects due to the cross-sectional study design.

Among participants with follow-up DEXA scans, a majority demonstrated stable or improved BMD, with only 19.4% showing a decrease. At baseline, 34.6% had osteopenia and 8.3% osteoporosis. The improvements observed may reflect the combined influence of AET with anti-resorptive therapies (calcium, vitamin D, bisphosphonates) and lifestyle advice, rather than AET alone. These associations emphasise the value of regular monitoring and early intervention in BC care, and suggest that proactive management may help maintain bone health and potentially reduce long-term fracture risk. Future studies should statistically adjust for confounders to better evaluate the impact of AET on bone density.

### 4.1. Strengths

This study employed a mixed-methods approach combining semi-structured telephone interviews with questionnaires, capturing both quantitative and qualitative insights into AET adherence. Participants could respond via QR code or prepaid envelope, achieving a 55% response rate, higher than the average 44.1% for similar surveys.

Use of the validated BMQ-AET questionnaire enhanced the reliability of adherence measurement. Open-ended questions provided qualitative insight beyond closed-ended responses. The inclusion criteria limited participants to those treated within the last five years, reducing recall bias while allowing adequate time on AET to assess adherence. Although cross-sectional, the medium-term follow-up of four years allowed the evaluation of adherence patterns and BMD trends. The participant cohort was diverse in age, education, ethnicity, gender, and socioeconomic background, supporting generalizability.

### 4.2. Limitations

Several limitations should be acknowledged. The small size of the non-adherent group (N = 13) limits the robustness of statistical comparisons. Non-response bias may have influenced results, as certain groups such as lower income and less educated individuals may be underrepresented.

The study was single-centre in the United Kingdom, which may limit generalizability due to geographic and demographic differences.

Self-reported data introduces potential biases, including recall bias, social desirability bias, and the Hawthorne effect, which may have led participants to overestimate adherence. Postal questionnaires risk misinterpretation, and Likert-scale questions may introduce extremity bias, though the 5-point scale was used to mitigate this.

Confounding factors remain a concern. Participants’ multiple medications, lifestyle interventions, and concurrent therapies may have influenced their experiences with AET and thus BMD outcomes. The associations observed cannot be interpreted as causal due to the cross-sectional study design.

### 4.3. Recommendations for Future Research

Future studies should include multiple centres and larger cohorts to enhance generalizability and allow deeper subgroup analysis. Longitudinal designs would better clarify how adherence evolves over time and identify factors influencing persistence. Incorporating face-to-face interviews may provide richer qualitative insight, particularly for older patients.

## 5. Conclusions

This study provides insight into adherence to AET and associated factors alongside trends in bone health. High adherence was observed overall, but side effects remain a key reason for discontinuation. Non-adherent participants demonstrated lower perceived necessity and higher concern regarding AET, highlighting the importance of healthcare professionals addressing patient beliefs to potentially improve adherence. Observed trends in BMD reinforce the value of proactive management, regular monitoring, and patient education. These findings inform clinical practice and suggest avenues for supporting adherence and bone health in patients on AET.

## Figures and Tables

**Table 1:** Comparison of BMQ-AET Necessity score between the adherent and non-adherent groups.

BMQ-AET Specific Necessity Score					
Patient Group	Number of Patients	Mean	Median	Standard Deviation	*p*-Value
NON-ADHERENT	13	12.08	12	2.69	<0.001 *
ADHERENT	143	19.22	20	3.17	

* Mann–Whitney U Test, r value (effect size) is 0.44.

**Table 2:** Comparison of BMQ-AET Concerns Score between the adherent and non-adherent groups within the study.

BMQ-AET Specific Concerns Score					
Patient Group	Number of Patients	Mean	Median	Standard Deviation	*p*-Value
NON-ADHERENT	13	17.00	17	2.68	0.002 *
ADHERENT	143	13.46	13	4.24	

* Mann–Whitney U Test, r value (effect size) is 0.25.

## Data Availability

No data is available due to privacy and confidentiality reasons.

## Abbreviations

List of abbreviations used as follows:

BC	Breast Cancer
AET	Adjuvant Endocrine Therapy
ER-positive	Oestrogen Receptor Positive
AI	Aromatase Inhibitor
GnRH	Gonadotropin Hormone-releasing Hormone
BMD	Bone Mass Density
DEXA	Dual-Energy X-ray Absorptiometry
BMQ-AET	Beliefs about Medicine Questionnaire–adjuvant endocrine therapy
